# A cortical astrocyte subpopulation inhibits axon growth *in vitro* and *in vivo*

**DOI:** 10.3892/mmr.2015.3702

**Published:** 2015-04-29

**Authors:** RUI LIU, ZHE WANG, LIN GOU, HANPENG XU

**Affiliations:** 1 Department of Physiotherapy and Rehabilitation, Tangdu Hospital, Fourth Military Medical University, Xi’an, Shaanxi 710038, P.R. China; 2 Unit of Spinal Surgery, Department of Orthopedic Surgery, Xijing Hospital, The Fourth Military Medical University, Xi’an, Shaanxi 710032, P.R. China; 3 Laboratory of Neuroimaging, Department of Neurology, UCLA School of Medicine, Los Angeles, CA 90002, USA

**Keywords:** astrocyte, heterogeneous, axon growth, dorsal root ganglia neurons

## Abstract

Astrocytes are the most heterogeneous and predominant glial cell type in the central nervous system. However, the functional significance of this heterogeneity remains to be elucidated. Following injury, damaged astrocytes inhibit axonal regeneration *in vivo* and *in vitro*. Cultured primary astrocytes are commonly considered good supportive substrates for neuron attachment and axon regeneration. However, it is not known whether different populations of cells in the heterogeneous astrocyte culture affect neuron behavior in the same way. In the present study, the effect of astrocyte heterogeneity on neuronal attachment and neurite outgrowth was examined using an *in vitro* and *in vivo* co-culture system. *In vitro*, neonatal cortical astrocytes were co-cultured with purified dorsal root ganglia (DRG) neurons and astrocyte growth morphology, neuron attachment and neurite growth were evaluated. The results demonstrated that the heterogeneous astrocyte cells showed two different types of growth pattern, typical and atypical. Typical astrocytes were supportive to neuron attachment and neurite growth, which was consistent with previous studies, whereas atypical astrocytes inhibited neuron attachment and neurite growth. These inhibitory astrocytes exhibited a special growth pattern with various shapes and sizes, a high cell density, few oligodendrocytes on the top layer and occupied a smaller growth area compared with typical astrocytes. Neurites extended freely on typical supportive astrocyte populations, however, moved away when they reached atypical astrocyte growth pattern. Neurons growing on the atypical astrocyte pattern demonstrated minimal neurite outgrowth and these neurites had a dystrophic appearance, however, neuronal survival was unaffected. Immunocytochemistry studies demonstrated that these atypical inhibitory astrocytes were glial fibrillary acidic protein (GFAP) positive cells. The existence of inhibitory astrocyte subpopulations in normal astrocytes reflects the complexity of the function of astrocyte populations. *In vivo*, DRG neurons in grey matter did not show neurite growth, while DRG neurons survived and showed robust axon outgrowth along the corpus callosum. In conclusion, further studies on this new type of inhibitory astrocyte subpopulation may deepen our understanding of the complex biology of astrocytes.

## Introduction

Astrocytes are the most predominant and heterogeneous glial cells in the central nervous system (CNS) (1,2). According to their morphology and location, astrocytes are classified into fibrous astrocytes of the white matter and protoplasmic astrocytes of the grey matter (3). Traditionally, astrocytes were viewed as a simple passive cell population able to support neurons and were deemed to be a homogeneous group of cells. However, in the late 19th century and the early 20th century Camillo Golgi and Ramon y Cajal noticed that although astrocytes share stellate features, their morphology is extremely diverse (4). Since then, several studies have demonstrated the morphological diversity of astrocytes *in vitro* and *in vivo* (5–7). Numerous studies have demonstrated that astrocytes are highly heterogeneous in terms of their gene expression profiles suggesting that astrocytes are functionally diverse (8–12).

Previous studies have demonstrated that astrocyte heterogeneity is not only apparent through cell morphology, but also gene expression (1,13). Even within a single brain region, astrocytes can be extremely different (1,8). Although the heterogeneity of astrocytes was documented over a century ago, the functional significance of this heterogeneity remains to be elucidated. This is particularly true with respect to neural damage and regeneration studies. The failure of neuron regeneration following injury in the adult mammalian CNS is considered to be the result of a decreased intrinsic regenerative capacity in affected neurons (14,15). The process is affected by numerous factors, including myelin-associated inhibitory factors (16,17), glial scars formed by damaged astrocytes and inhibitory molecules secreted by damaged astrocytes (18,19).

Damaged astrocytes form mechanical and molecular barriers for axon growth *in vivo* and *in vitro* (20,21). By contrast, primary cortical astrocytes have been considered a good supportive substrate for neuron growth (22). This astrocyte culture also alters the inhibitory effect of oligodendrocytes (23). These observations raised the following question: Do these different populations of astrocyte cells have the same effect on neuron behavior? It is highly possible that astrocytes are a functionally heterogeneous population of cells, although experimental evidence for the functional heterogeneity of astrocytes is scarce (12).

The present study aimed to address this question by co-culturing purified dorsal root ganglia (DRG) neurons with primary cortical glial cells. According to their effects on neuron attachment and neurite growth, two astrocyte subpopulations were identified. A subpopulation, which supported neuron attachment and neurite outgrowth, termed typical supportive astrocytes, and a subpopulation, which inhibited neuron attachment and neurite outgrowth, termed atypical inhibitory astrocytes. This inhibitory astrocyte subpopulation may be important in the failure of neuron regeneration following injury. Further investigation on these inhibitory astrocytes may deepen our understanding of astrocyte heterogeneity and their role in the development and maturation of the nervous system.

## Materials and methods

### Animals

A total of 24 E14 Sprague Dawley (SD) rats and rat pups (P2-5) were obtained from the Fourth Military Medical University animal facility (Xi’an, China). All animal experimental protocols were approved by the Fourth Military Medical University Animal Committee and were performed in accordance with the guidelines of the National Institutes of Health (Bethesda, MD, USA). All efforts were made to minimize the number of animals used and their suffering.

### Cell culture reagents and antibodies

Fetal bovine serum (FBS) was obtained from HyClone (Logan, UT, USA). Dulbecco’s modified Eagle’s medium (DMEM), DNase, trypsin-EDTA, neurobasal medium and B27 serum-free supplement were obtained from Invitrogen Life Technologies (Carlsbad, CA, USA). 2.5S neural growth factor (NGF), laminin, polylysine and fluorodeoxyuridine (FudR) were purchased from Sigma-Aldrich (St. Louis, MO, USA). The following primary antibodies were obtained from Abcam (Cambridge, MA, USA): Mouse monoclonal anti-neurofilament [NF; immunoglobulin (Ig)G2a; cat. no. ab128824; 1:1,000], rabbit polyclonal anti-p75 neurotrophin receptor (IgG; cat. no. ab8874; 1:1,000), mouse monoclonal anti-S100 (IgG2a; cat. no. ab4066; 1:1,000), rabbit polyclonal anti-NG2 (IgG; cat. no. ab101807; 1:1,000), mouse monoclonal anti-microglia cell specific OX42 (IgG2a; cat. no. ab1211; 1:500), rabbit polyclonal anti-fibronectin (IgG; cat. no. ab23751; 1:1,000) and mouse monoclonal anti-calcitonin gene related peptide (CGRP; IgG; cat. no. ab10987; 1:500). Mouse monoclonal anti-oligodendrocyte marker O4 antibody (IgM; cat. no. NL1326V; 1:50) was purchased from R&D Systems (Minneapolis, MN, USA). Mouse monoclonal anti-galactocerebroside antibody (GalC; IgG3; cat. no. MAB342; 1:50) was purchased from EMD Millipore (Boston, MA, USA). Rabbit polyclonal anti-glial fibril-lary acidic protein antibody (IgG; GFAP; cat. no. Z0334l 1:1,000) was obtained from Dako (Copenhagen, Denmark). Alexa Fluor^®^ 594 goat anti-rabbit IgG polyclonal antibody (cat. no. A-11037, 1:500), Alexa Fluor^®^ 488 rabbit anti-mouse IgG polyclonal antibody (cat. no. A-11059; 1:1000), Alexa Fluor^®^ 594 goat anti-mouse IgG polyclonal antibody (cat. no. A-11032; 1:1000), Alexa Fluor^®^ 488 goat anti-rabbit IgG polyclonal antibody (cat. no. A-11008; 1:500) and Alexa Fluor^®^ 594 donkey anti-mouse IgM polyclonal antibody (cat. no. A-21044, 1:50) were purchased from Life Technologies (Carlsbad, CA, USA).

### Primary glial cell culture

Neonatal SD rat pup cortices were isolated and all meningeal membranes were carefully removed completely under a stereomicroscope (SZX16-3136; Olympus Corporation, Tokyo, Japan). Tissue was washed several times in DMEM medium and cut into small sections (1 mm^3^) and incubated in 0.125% trypsin-0.05% EDTA for 15 min at 37°C. DMEM medium containing 10% FBS was added to terminate digestion. Single cell suspensions were obtained by triturating through fire polished Pasteur pipettes and washed twice with 10% FBS DMEM medium containing DNase (10 *µ*g/ml). Cells were seeded in polylysine coated 6-well plates or 75 cm^2^ flasks at 1.0×10^4^/cm^2^. In immunocytochemistry experiments, cells were seeded at the same density on 12 mm polylysine coated glass coverslips and cultured in 4-well plates. Glial cells were maintained in 10% FBS DMEM medium at 37°C with 5% CO_2_. The medium was refreshed every 2 days by removing half and relacing it with fresh medium. After 20 days, cells reached confluence and were ready for the addition of DRG neurons. In pilot experiments, another purification step was performed according to McCarthy and de Vellis (24). The results did not show any difference, thus in the following experiments, this purification step was omitted.

### DRG neuron culture purification

Pure DRG neuron cultures were generated as described previously (25). Briefly, SD rats (E14) were euthanized by CO_2_ overdose. DRG neurons were isolated, pooled and digested in 0.125% trypsin-0.05% EDTA at 37°C for 15 min. Single cell suspensions were obtained by the same method as glial cells. Following washing twice with DMEM containing 10% FBS and 10 *µ*g/ml DNase, cells were seeded at 0.5–1.5×10^4^/cm^2^ on laminin-coated coverslips in normal NBG medium (neurobasal medium with 2% B27 and 50 ng/ml NGF) and stored at 37°C in a 5% CO_2_ incubator. After 15 h, stock FudR was added to the NBG medium to a final concentration of 20 *µ*M and kept for 72 h. Subsequently, FudR medium was changed to NBG medium and the medium was changed by half every 2 days. After 10 days, the cells were ready for co-culture.

### Glial DRG neuron co-culture

When glial cells reached confluence (~20 days after seeding), DRG neurons (~10 days after seeding) were collected, dissociated and seeded at 4.0×10^3^/cm^2^ on confluent glial cells in 6-well plates and 4-well coverslips. Co-cultures were maintained in 10% FBS DMEM containing NGF (50 ng/ml) at 37°C and 5% CO_2_. Different cell growth pattern areas were recognized and recorded under a phase contrast microscope (Ortholux II; Leitz, Wetzlar, Germany).

### Neuron attachment and neurite growth assay

Images of the DRG and glial cell co-culture were captured 72 h after DRG addition. DRG neuron growth parameters, including neuronal density, neurite outgrowth (neurite length >5 neuron diameters) and neurites with dystrophic end-bulbs on typical and atypical areas were calculated, respectively. Nuclei were counterstained with 4′-6-diamidino-2-phenylindole (DAPI; Life Technologies).

### Immunocytochemistry

Cultures were fixed with 4% paraformaldehyde in 0.01 M PBS for 10 min at room temperature. Following washing three times with 0.01 M PBS, cultures were blocked by 0.01 M PBS containing 10% normal goat serum and 0.01% Triton X-100 for 1 h (for oligodendrocyte staining the Triton X-100 treatment step was omitted). Coverslips were incubated with primary antibodies at 4°C overnight. Following rinsing with 0.01 M PBS, cells were incubated with a mixture of secondary antibodies for 1 h at room temperature.

### Quantitative calculation

Images were acquired using an Olympus IX70 microscope (Olympus, Tokyo, Japan). All the data were collected and repeated at least four times. Images were assayed using Image J v3.91 software (National Institutes of Health) and analysis of variance was used for statistical analysis. In every culture, data are presented as the mean ± standard error of the mean. Cell density in the typical and atypical cell growth area were calculated by counting the number of DAPI^+^ nuclei in at least 10 randomly selected visual fields (magnification, ×100). Oligodendrocyte density on the typical and atypical cell growth areas were calculated by counting O4^+^ oligodendrocyte precursors and GalC^+^ mature oligodendrocyte cells in at least 10 randomly selected visual fields (magnification, ×100). The atypical growth area percentage in the total growth area was calculated by measuring all atypical growth areas in one culture and the typical area percentage was calculated using the following formula: Total growth area - atypical growth area / total growth area. NF^+^ DRG neurite number, neurite morphology and dystrophic endbulbs were examined on typical and atypical astrocyte patterns in at least 10 randomly selected visual fields (magnification, ×100). GFAP^+^ astrocytes were calculated on typical and atypical astrocyte patterns in a similar manner.

### In vivo DRG transplantation and axon growth

DRG neurons were dissected by microsurgery under sterile conditions from E14 SD rats. DRG neurons were digested in trypsin-EDTA (0.125% trypsin with 0.05% EDTA) at 37°C for 20 min and digestion was terminated by 20% FBS in DMEM. Single cell suspensions were obtained by aspirating up and down through a fire polished Pasteur glass pipette. The cell density was adjusted to 1×10^3^ cells/*µ*l in L15 medium. In total, eight adult SD rats were used in this experiment. To declare the injection site coordination on the rat skull surface, Bregma point was set as 0, anterior Bregma as positive and posterior Bregma as negative as described previously (26). Cell suspensions (0.5 *µ*l; 5,000 DRG neurons) were injected into either left cortical grey matter (anterior-posterior direction, posterior Bregma point 0.5 mm; middle-lateral from middle line, lateral 3.0 mm from middle line; depth from brain surface into cortex 2.0 mm) or the right corpus callosum (anterior-posterior direction, posterior Bregma point 0.5 mm; middle-lateral from middle line, lateral 3.0 mm from middle line; depth from brain surface into cortex 3. 0mm) of adult SD rats. At 3 weeks after transplantation, animals were perfusion fixed with 4% paraformaldehyde in 0.01 M PBS and 20 *µ*m cryostat brain sections were stained using anti-calcitonin gene related peptide (CGRP) and GFAP antibodies. Neuron survival and axon outgrowth within the grey matter and white matter were calculated. In total, eight animals were analyzed in each group.

## Results

### Confluent astrocyte enriched culture exhibits distinct growth patterns

**Figure 1:**
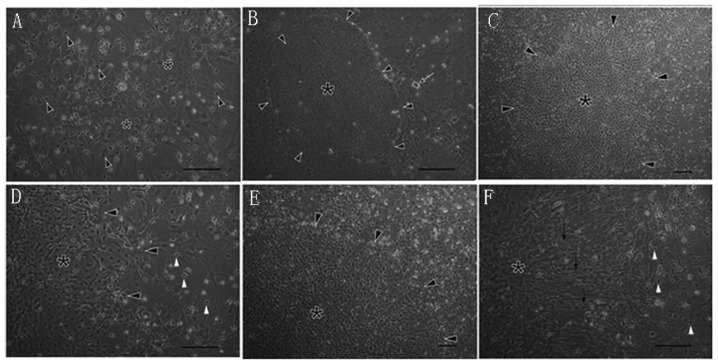
Different cortical glial cell growth patterns coexist in the same culture. (A) Typical growth pattern with two cell layers. Arrows indicate the small dark, process-bearing top layer cells and stars indicate the light, flat, fibroblast-like bed layer cells. (B) Atypical pattern. The star indicates a round cobblestone-like high compact atypical growth pattern and arrowheads indicate the boundary between the typical and atypical pattern. The arrow indicates one dorsal root ganglia neuron with an extending axon. (C and D) Atypical pattern. The star indicates an irregular high density atypical pattern and arrowheads indicate the boundary between atypical and typical growth patterns. (D) White arrowheads indicate the small dark cells on the typical astrocyte growth pattern, which are absent from the atypical pattern. (E and F) Third atypical pattern. The star indicates the polarized arranged atypical growth pattern. Arrowheads indicate the boundary between the typical and atypical growth patterns. Black arrows indicate the radially oriented astrocytes and white arrowheads indicate the small dark cells on the typical pattern, which are absent from the atypical pattern. Scale bar, 100 *µ*m. All atypical patterns are composed of 100–1,000 clustered cells with a similar morphology.

**Figure 2:**
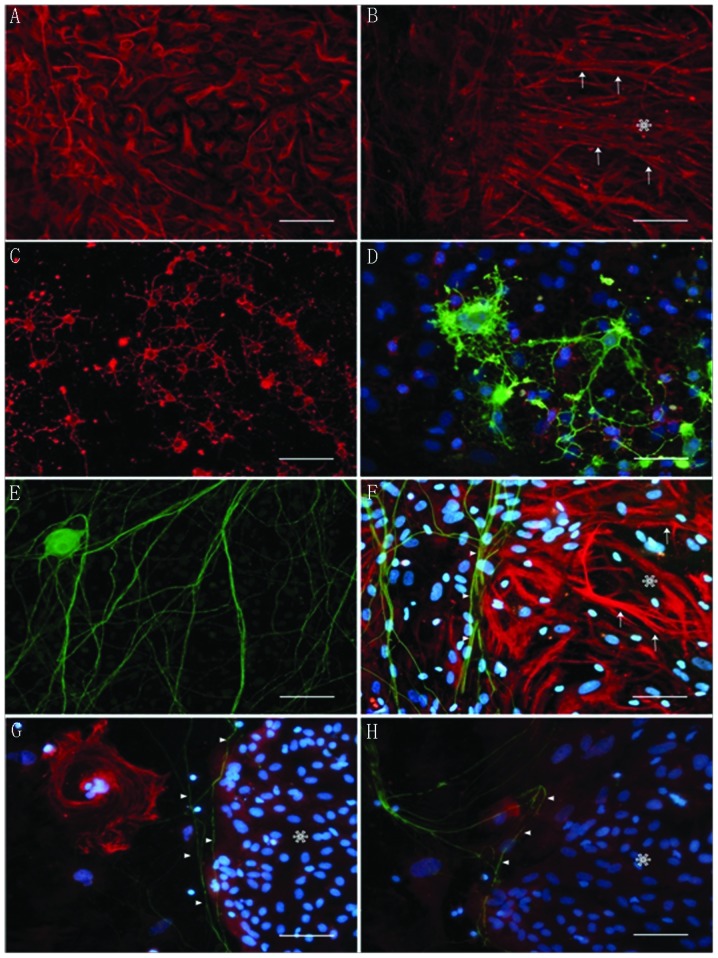
Immunocytochemistry identification of glial cells on typical and atypical growth patterns. (A) GFAP^+^ glial cells on the typical growth pattern show different shapes and a random arrangement. (B) Local details of one polarized arranged atypical pattern. The star indicates the atypical pattern; white arrows indicate the polarized orientation of GFAP^+^ cells. (C) Typical pattern top layer O4^+^ cells showing the morphology of oligodendrocyte precursors. (D) Typical pattern top layer GalC^+^ cells (green). Red (GFAP^+^) indicates astrocytes and blue indicates the nuclei of astrocytes. (E) NF^+^ neurites on the typical growth pattern show a freely extending network around DRG neurons. (F) NF^+^ neurites (green) extending along the boundary of the typical pattern and polarized arranged atypical pattern (star); white arrows indicate polarized GFAP^+^ cells (red); arrowheads indicate the neurites avoiding crossing into the atypical pattern. (G) NF^+^ neurites (green) on the typical pattern extend along the boundary of the cobblestone-like atypical pattern. White arrows indicate neurites and the star indicates the GFAP^+^ cobblestone-like atypical pattern with a high cell density. (H) NF^+^ neurites (green) from the typical pattern move away from crossing into the GFAP^+^ (red) cobblestone-like atypical pattern. White arrows indicate neurite direction changes. Scale bar=50 *µ*m. Red, Alexa Fluor^®^ 594-conjugated secondary antibodies; green, Alexa Fluor^®^ 488-conjugated secondary antibodies; blue, DAPI staining. GFAP, glial fibrillary acidic protein; DRG, dorsal root ganglia; NF, neurofilament; GalC, galactocerebroside.

**Figure 3:**
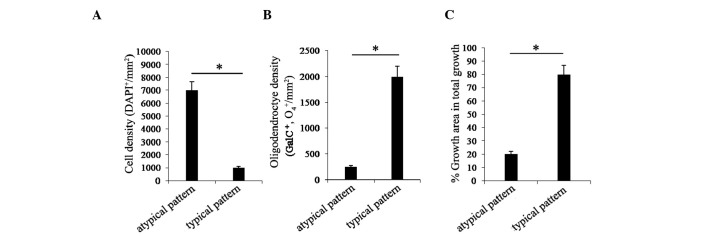
Quantitative comparison of glial cells in different growth patterns. (A) Astrocyte cell density of the bottom layer in the typical and atypical growth patterns (^*^P<0.001). (B) Top layer (O4^+^ and GalC^+^) cell density in the typical and atypical growth patterns (^*^P<0.001). (C) Growth area of typical and atypical astrocyte patterns (^*^P<0.001). Values are presented as the mean ± standard error of the mean of four independent cultures. ^*^P<0.001, as compared with the atypical pattern. Scale bar=100 *µ*m. GalC, galactocerebroside; DAPI, 4′,6-diamidino-2-phenylindole.

Following plating at a low density, cortical glial cells proliferated, expanded and at 20 days, a confluent astrocyte enriched culture was obtained. Under a phase contrast microscope (BX45-72P15; Olympus Corporation), typical astrocyte glial cell morphologies were observed as McCarthy and Vellis reported (21), including a confluent glial culture formed by two cellular layers. The top layer was composed of dark, small and process-bearing cells and the bottom layer was composed of light, large, fibroblast-like polygonal cells with prominent nuclei and obscure profiles, and glial cells grew in a random orientation pattern (Fig. 1A). Immunocytochemistry staining demonstrated that the bottom cells were astrocytes (>95% GFAP^+^; Fig. 2A and B) and the top layer cells were composed of immature (>95% O4^+^; Fig. 2C) and some mature (CalC^+^; Fig. 2D) oligodendrocytes. Typical astrocytes occupied the majority of the growth area (>70% of the total growth area; Fig. 3C). Besides these typical astrocyte growth patterns, certain atypical astrocyte growth patterns were also observed, which occupied a minor growth area (<30% total growth area; Fig. 3C). These atypical growth patterns could be easily discriminated from typical ones by several unique characteristics: These glial cells usually grew together and formed homogenous cell clusters with few or no oligodendrocytes on top of them (Figs. 1B–D and 3B) and were usually small, with a high cell density and grew in random orientation (Figs. 1B–D and 3A). Certain atypical glial cells demonstrated a spindle-shaped morphology and polarized orientation growth patterns (Figs. 1E, F and 2B). Immunocytochemistry demonstrated that these atypical cells were also GFAP^+^ (Fig. 2B, F, G and H). The high density and compact growth pattern of atypical glial cell clusters formed separated special growth areas with cells of various sizes (between 10 *µ*m and 10 mm, composed of 100–10,000 cells) and shapes (from nearly round to irregular). They distributed randomly within the relatively homologous typical astrocytes giving the whole glial culture a mosaic appearance.

The differences between typical and atypical astrocyte subpopulations were quantified and shown in Fig. 3A, B and I. The average cell density of typical astrocytes was significantly lower than atypical astrocytes (Fig. 3A; P<0.001), however, the density of oligodendrocytes (O4^+^ and GalC^+^) on top of typical astrocytes was significantly higher than atypical astrocytes (Fig. 3B; P<0.001). The percentage growth area of typical astrocytes was significantly higher than atypical astrocytes (Fig. 3C; P<0.001).

### Heterogeneous astrocyte growth patterns exert different supportive properties on neurite growth and neuron attachment

The coexistence of different growth patterns of typical and atypical astrocytes in the same glial culture may, to a certain degree, reflect the heterogeneity of astrocytes. However, the functional significance of this heterogeneity remains to be elucidated. Previous studies have demonstrated that typical astrocytes prepared using the technique performed by McCarthy and Vellis formed a good supportive substrate for neuron attachment and growth (27). The coexistence of typical and atypical astrocytes provided us with the opportunity to investigate the effect of astrocyte heterogeneity on neurons. For this purpose, high purified DRG neurons were added onto confluent glial cultures and neuronal attachment and growth were evaluated in a qualitative and quantitative way. Within 2 h following addition, DRG neurons settled on top of typical and atypical astrocytes as single or small clusters (>3) with a similar density and this loose attachment could be disrupted by partial disturbance of the culture medium.

**Figure 4:**
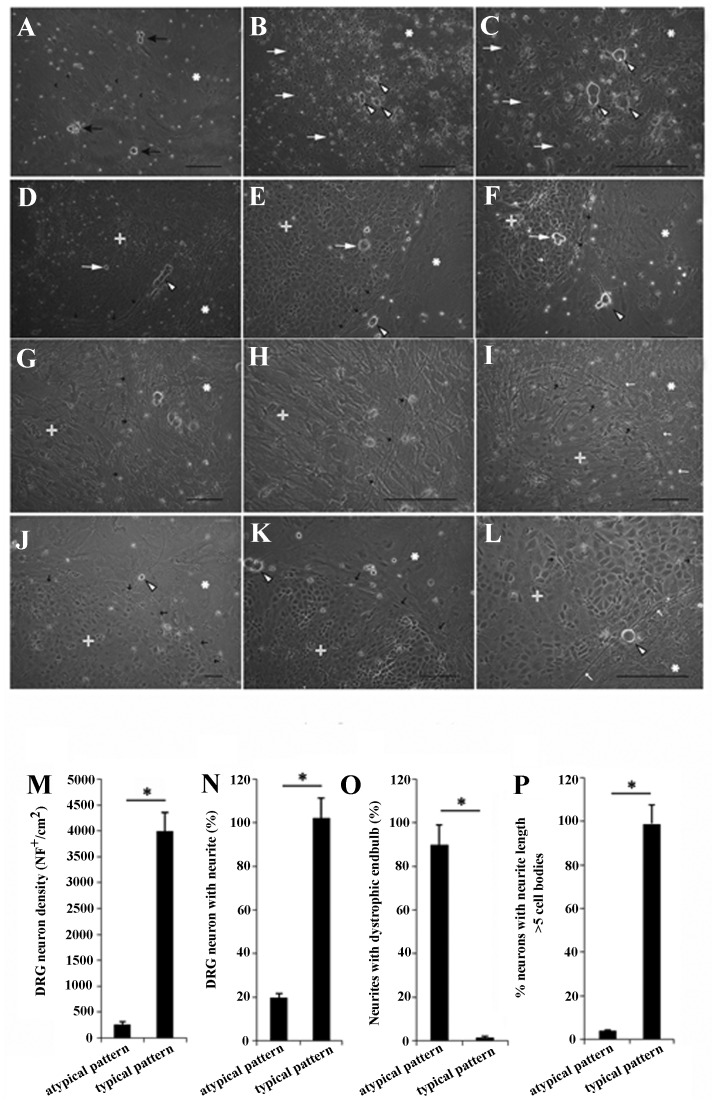
Different astrocyte growth patterns affect DRG neuron attachment and neurite growth. (A–C) DRG neurons attached and neurites grew on the typical pattern. (A) The star indicates bottom layer cells, black arrows indicate the DRG neurons and small black arrows indicate the neurite network between DRG neurons. (B and C) The star indicates bottom layer cells, arrowheads indicate DRG neurons and white arrows indicate top small dark cells. (D–K) DRG neurons attached and neurites grew on the atypical pattern. (D) White cross indicates the atypical pattern, the white star indicates the typical pattern and small black arrows indicate the boundary between typical and atypical patterns. Arrowheads indicate DRG neurons at the boundary with dense neurites selectively growing on the typical pattern. The white arrow indicates single neuron attachments on the atypical pattern without axon outgrowth. (E) The white cross indicates a cobblestone atypical pattern, the white star shows the typical pattern and small black arrows indicate neurites extending along the boundary of these two patterns. Arrowheads indicate a DRG neuron on the typical pattern with neurites extending freely. The white arrow shows one single DRG neuron on the atypical pattern without neurite outgrowth. (F) The white arrow indicates three DRG neurons with single thick, short and stiff neurites on the cobblestone-like atypical pattern and the small white arrow indicates the dystrophic endbulb of the neurite. Arrowheads indicate the DRG neurons on the typical growth pattern with branched neurites. (G and H) Neurites grew along the boundary between the typical and polarized atypical pattern. (I) Neurites take shortcut straight pathway to cross over the atypical pattern from the typical pattern. Small white arrows indicate neurites extending along the typical and atypical pattern boundary; black small arrows indicate the neurite shortcut. (J and K) Neurites on the typical pattern altered direction and avoided crossing into the atypical pattern. The white cross indicates the cobblestone-like atypical pattern. The white star indicates the typical pattern; the arrowhead indicates DRG neurons; small black arrows indicate neurites that moved away from crossing into the atypical area and selectively extended on the typical pattern along the boundary between them. (L) Neurite end tips showed dystrophic endbulbs. The white cross indicates the cobblestone-like atypical pattern; the star indicates the typical pattern. The arrowhead indicates a DRG neuron. White small arrows show neurites extending along the boundary of the typical and atypical pattern. Black small arrows indicate dystrophic endbulbs of short, straight neurites crossing into the atypical pattern. (M) DRG neuron attachment on the typical and atypical pattern (^*^P<0.0001). (N) DRG neurons with neurites on the typical and atypical growth pattern (^*^P<0.001). (O) DRG neuron with neurite (neurite length >5 cell bodies; ^*^P<0.001). (P) Neurite with dystrophic endbulbs on the typical and atypical pattern (^*^P<0.001). Values are presented as the mean ± standard error of the mean of four independent cultures. ^*^P<0.001, as compared with the atypical pattern. Scale bar=100 *µ*m. DRG, dorsal root ganglia; NF, neurofilament.

For the typical supportive astrocytes, at 6 h after the addition of the neurons, the neuron attachment was tight and washing under a medium flow did not allow resuspension of the neurons. The majority of neurons initiated neurite outgrowth at this stage and certain neurites extended as long as three cell bodies. After 24 h, neurite length reached as long as five cell bodies and at 48 h, the robust outgrowing neurites had formed a network by connecting with each other and at this stage it was difficult to discern individual neurites (Fig. 4A). Neurite growth on typical astrocytes was not inhibited by oligodendrocytes and they grew in a smooth and flexible way through oligodendrocytes without signs of extra constraints or abrupt trajectory changes. Even in areas with a high density of oligodendrocytes, the extending neurite tips demonstrated no dystrophic end bulbs (Fig. 4B and C).

In contrast to typical astrocytes, atypical astrocytes demonstrated different effects on DRG neurons. At 6 h after addition, the majority of settled neuron attachments remained relatively weak and they detached from the bottom astrocytes following partial medium disturbance. After 24 h, less than half of the neurons initiated neurite growth and the length of neurites was less than three cell bodies. In certain areas, neurons had no neurite growth after 72 h. Neurons could maintain a normal morphology without neurite growth even after 7 days and had no signs of cellular degeneration or death (Fig. 4D and E). Neurites in the atypical area demonstrated a short, thick, stiff and straight appearance and were limited within the atypical area, their terminals usually ending with dystrophic end bulbs (Fig. 4F).

At the boundary of typical and atypical areas, the dense neurites growing on the typical area usually avoided penetrating into the neighboring neurite sparse atypical area (Fig. 4G and H; Fig. 2F). Individual neurites altered their growth course abruptly on approaching the atypical area and selectively grew along the boundary on typical astrocytes (Fig. 4J and K; Fig. 2G and H). When short neurites from typical astrocyte areas grew into the atypical area, their morphology altered from a flexible curve thread-like morphology to a stiff straight one (Fig. 4I). In addition, neurites stopped growing with dystrophic end bulbs terminals (Fig. 4L).

Quantitative assessment of the different neuron attachments and neurite growth on typical and atypical areas are shown in Fig. 4M–P. Neuron attachment on the typical area was significantly higher than on atypical astrocytes (Fig. 4M; P<0.001) 72 h after seeding. Neurite initiation on atypical astrocytes was markedly delayed (only 20% of neurons initiated neurite growth) and on typical atrocytes, ~100% showed neurite growth (Fig. 4N; P<0.001). The majority of neurites on atypical astrocytes showed dystrophic end bulbs but few were found on typical astrocytes (Fig. 4O; P<0.001). The percentage of neurons with neurites longer than five cell bodies on atypical astrocytes was significantly lower than typical astrocytes (Fig. 4P; P<0.001). Immunocytochemistry results demonstrated that typical and atypical astrocytes were GFAP^+^ and neurites grew along their boundary or moved away from crossing into the atypical astrocytes (Fig. 2F–H).

### Transplanted DRG neurons show different neurite growth behavior in cortical grey matter and white matter

**Figure 5:**
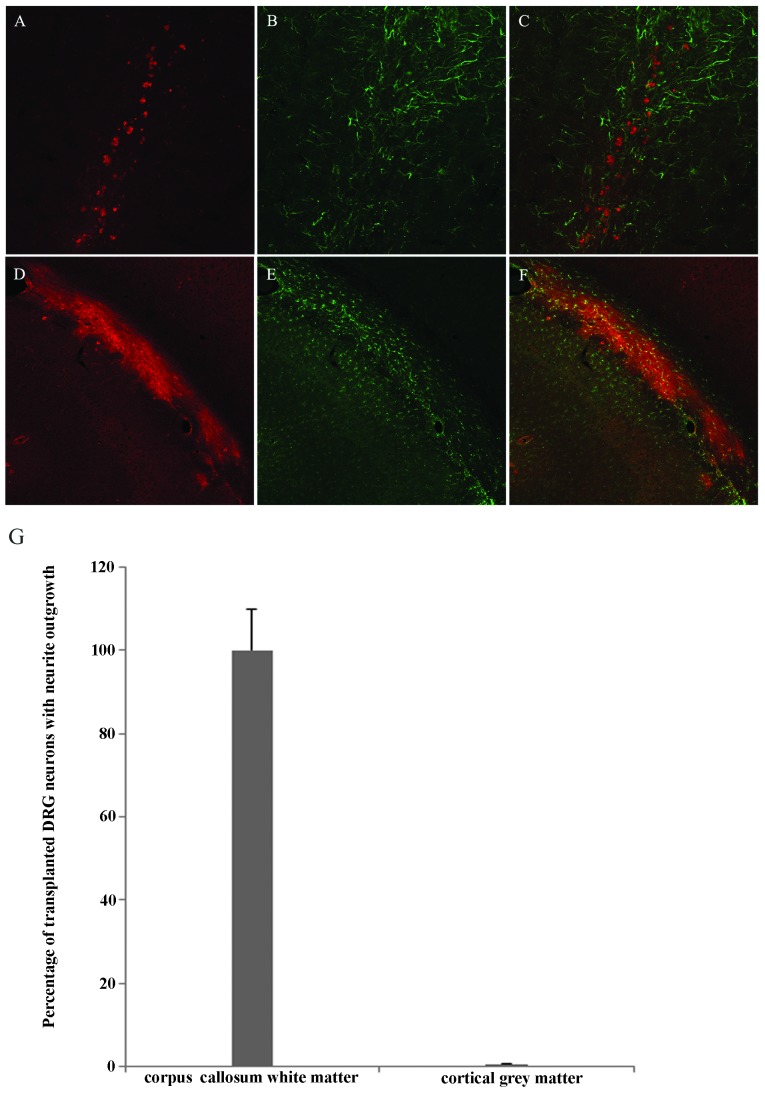
Transplanted DRG neurons show different neurite growth behavior in cortical grey matter and white matter. (A) CGRP^+^ DRG neurons (red) on cortical grey matter exhibit minimal axon outgrowth. (B) GFAP^+^ astrocytes (green) grow on cortical grey matter. (C) DRG neurons show minimal axon outgrowth on astrocytes in cortical grey matter. (D) CGRP^+^ DRG neurons and fibers (red) on cortical white matter exhibit robust axon outgrowth. (E) GFAP^+^ astrocytes (green) grow on the corpus callosum. (F) DRG neurons show robust axon outgrowth on astrocytes in cortical white matter. Red, Alexa Fluor^®^ 594-conjugated secondary antibodies; green, Alexa Fluor^®^ 488-conjugated secondary antibodies Scale bar=50 *µ*m. (G) Neonatal rat DRG neurons were transplanted into brain grey (cortex) and white matter (corpus callosum) and 3 weeks later, the neurite outgrowth of transplanted DRG neurons was detected using the CGRP antibody. All the transplanted DRG neurons in the corpus callosum demonstrated extensive neurite outgrowth, however, no observable neurite outgrowth was detected in DRG neurons transplanted into grey matter (P<0.0001; t-test). DRG, dorsal root ganglia; GFAP, glial fibrillary acidic protein; CGRP, calcitonin gene related peptide.

Using the microinjection method, purified DRG neuron single suspensions were transplanted into cortical grey matter and the underlying corpus callosum white matter. The growth of neurites in these two regions was significantly different. DRG neurons in the grey matter did not show neurite growth, neuron survival was clearly observed in the injection site and no significant neurite growth was observed. Grey matter astrocytes around the injection site demonstrated a mild astrogliosis response to injection (Fig. 5A–C). By contrast, transplanted CGRP^+^ DRG neurons survived and demonstrated robust axon outgrowth along the corpus callosum. White matter astrocytes around the injection site demonstrated a mild astrogliosis response to injection (Fig. 5D–F). All the transplanted DRG neurons in the corpus callosum demonstrated extensive neurite outgrowth, but no observable neurite outgrowth was detected in DRG neurons transplanted into grey matter (Fig. 5G).

## Discussion

Astrocytes are the most abundant glial cells in the CNS. Recent advances in astrocyte research have markedly altered our understanding of them (28). *In vivo* and *in vitro* evidence indicates that astrocytes are an extremely heterogeneous population based on their morphology, antigenicity, receptors, channels, growth factors, synaptic transmission and responses to brain-blood regulation (1,28,29).

This heterogeneity reflects the diverse functions of astrocytes during the development and maturation of the nervous system. During development, transient glial cell boundaries are found to restrict and guide axonal pathway finding processes (30–32). It is well established that damaged astrocytes inhibit neurite growth *in vivo* and *in vitro* (21,33,34), however, in normal culture conditions, astrocytes form a good supportive substrate for neurite growth and alter the inhibitory effect of oligodendrocytes on neurite regeneration (20,21). Few studies have noted the effects of astrocyte heterogeneity on neurite growth (35–37).

These earlier studies prompted us to examine the effects of astrocyte heterogeneity on neuron growth using purified DRG neurons with neonatal cortical mixed glial cell cultures. Our results demonstrated that at least two astrocyte types coexist in primary cortical astrocyte cultures. The first type were typical astrocytes, which formed the majority of the culture area with oligodendrocytes growing on top. These astrocytes supported DRG neuron attachment and axon growth without inhibition and their growth pattern was consistent with numerous previous studies (38,39). The second astrocyte subpopulation was composed of atypical astrocytes, which formed a small culture area with few oligodendrocytes growing on top and had a high cell density and polarized orientation. These atypical astrocytes exerted strong inhibitory effects on neuron attachment and neurite outgrowth without affecting neuron survival. When glial cells are cultured using the method by McCarthy and Vellis, typical astrocytes dominate the confluent cells and no atypical astrocyte substructures are formed.

Several modifications of the classical McCarthy and Vellis method in the present study may contribute to the atypical astrocyte findings. To reduce earlier cell to cell contact inhibition, cortical cells were plated at a low density (1.0×10^4^/cm^2^ instead of 5.0–10×10^4^/cm^2^) to prolong glial precursor cell proliferation and expansion period before they reach confluence. To maintain their intact state and heterogeneity as much as possible, purification steps were omitted (this omission did not change the results), without the purification step, astrocyte purity was verified by GFAP to be >95% with few contaminating cells. To minimize the effects from other cell types during co-culture and monitor neuron behavior at a large scale (100–1,000 cell cluster), highly purified DRG (99%) neurons were selected to co-culture with astrocytes due to their well-established strong intrinsic regeneration capacity, long neurite growth and the fact that they are easily distinguished under the microscope (14,40,41).

Atypical astrocytes affected neurons in numerous ways, including inhibiting neuron attachment, inhibiting neurite growth initiation, retarding axon growth and provided poor neurotrophic support, however, they had no effect on cell survival. Since the atypical inhibitory astrocytes are enclosed by supportive typical astrocytes, the localized inhibitory effects implied that the inhibition may be mediated by cellular membrane associated molecules or short distance effect molecules.

Astrocytes cultured from different brain regions using the method performed by McCarthy and Vellis have been broadly used in glial neuron interaction studies (24,42,43). They form a good substrate for various neurons and support neuron attachment and neurite growth (23,39,44,45). *In vivo* experiments in the present study also confirmed this. In the present study, the mild astrocyte response at the injection site indicated that the injury-induced glial response does not account for the axon outgrowth pattern difference. Fibrous astrocytes are supportive for axon growth, however, the protoplasmic astrocytes in grey matter may be inhibitory to axon growth. Based on several characteristics of the inhibitory astrocytes, it was hypothesized that they may represent a distinct astrocyte subtype for the following reasons: i) These cells express GFAP and usually grow together to form certain substructures with a similar morphology, which implies they are derived from a common precursor cell; ii) these cells form inhibitory substructures that are stable for up to 2 months (the longest time point in the present study) without alterations (such as changing to supportive flat cells); iii) neurons growing on them could survive for a long period of time (2 months) without neurite extension and degeneration; iv) following a long culture period (2 months), these cells maintained their inhibitory effects on newly added DRG neurons (data not shown); v) fibroblast as well as other cell type (oligodendrocytes and microglia cells) markers were not expressed on inhibitory astrocytes; vi) these cells demonstrated high mobility following injury (data not shown).

As supplement to the current opinion about astrocytes, the results of the present study indicated that heterogeneous astrocytes are not homologous in their effects on neuron behavior. A subpopulation of astrocytes exerted inhibitory effects on neuron attachment and axon growth under normal culture conditions. Elucidating the nature of these inhibitory astrocytes may deepen our understanding of the complex glial environment in normal and pathological conditions and our co-culture model may also aid in identifying inhibitory astrocytes and the underlying molecular mechanisms, as well as in developing treatments that may improve axon regeneration.
